# NMDA Receptors in Health and Diseases: New Roles and Signaling Pathways—Anti-N-Methyl-D-Aspartate Receptor (NMDAR) Autoantibodies as Potential Biomarkers of Fatigue in Patients with Rheumatic Diseases

**DOI:** 10.3390/ijms24043560

**Published:** 2023-02-10

**Authors:** Tatjana Marinoska, Tamara Möckel, Konstantinos Triantafyllias, Sebastian Boegel, Matthias Dreher, Felix Luessi, Andreas Schwarting

**Affiliations:** 1 Center for Rheumatic Disease Rhineland-Palatinate, 55543 Bad Kreuznach, Germany; 2 Division of Rheumatology and Clinical Immunology, University Medical Center of the Johannes Gutenberg University of Mainz, 55131 Mainz, Germany; 3 University Center for Autoimmune Disease, University Medical Center of the Johannes Gutenberg University of Mainz, 55131 Mainz, Germany; 4 Division of Neurology, University Medical Center of the Johannes Gutenberg University of Mainz, 55131 Mainz, Germany

**Keywords:** anti-NMDAR2 antibodies, fatigue, rheumatic diseases, neurofilament light chain

## Abstract

Fatigue is a widespread and complex symptom with motor and cognitive components; it is diagnosed predominantly by questionnaire. We recently published a correlation between anti-N-methyl-D-aspartate receptor (NMDAR) antibodies and fatigue in patients with SLE (systemic lupus erythematosus). In the present study, we examined whether this association also applies to patients with other rheumatic diseases. Serum samples of 88 patients with different rheumatic diseases were analyzed for the presence of anti-NR2 antibodies and Neurofilament light chain (NfL) protein. The severity of fatigue was determined according to the FSMC questionnaire (Fatigue Scale for Motor and Cognitive Functions) and correlated with the circulating antibody titer and NfL level accordingly. Positive titers of anti-NR2 antibodies were detected in patients with both autoimmune and non-autoimmune rheumatic diseases. These patients suffer predominantly from severe fatigue. The circulating NfL level did not correlate with the anti-NR2 titer and the fatigue severity in all patient groups. The association of severe fatigue with circulating anti-NR2 antibodies in patients with rheumatic diseases, independently from the main disease, suggests an individual role of these autoantibodies in fatigue pathophysiology. Thus, the detection of these autoantibodies might be a helpful diagnostic tool in rheumatic patients with fatigue.

## 1. Introduction

Fatigue is one of the most debilitating and frequent symptoms in rheumatic patients. It is a common feature of cancer and many chronic inflammatory and immunological diseases and is particularly prominent in rheumatological conditions. The prevalence of fatigue varies within rheumatic diseases; it is estimated as lowest in patients with osteoarthritis (35%) and highest in patients with fibromyalgia (82%) [1]. In contrast, the prevalence of fatigue in the general population defined as chronic fatigue syndrome ranges between 0.007 and 2.8% [2].

The clinical significance of chronic fatigue in patients with inflammatory rheumatic diseases is underlined by its association with lower self-reported health-related quality of life (QoL). While the complications of fatigue are not life-threatening, its chronicity can lead to severe debility, markedly affecting mood, sleep, routine daily activities, work capacity and employment opportunities [3]. Rheumatic patients rank fatigue as one of their most disabling symptoms, even when inflammatory disease activity is under control [2]. 

In contrast to the clinical importance of fatigue in rheumatic diseases, its pathology remains poorly understood. No biomarkers or experimental tests for objective measurement of fatigue are yet available. Thus, self-report questionnaires continue to be the diagnostic gold standard, with many conceivable and established limitations. 

The pathogenesis of fatigue, particularly in the setting of inflammatory rheumatic diseases, is not yet completely elucidated, but growing evidence suggests a multifaceted picture [3]. An association has been described with various psychological and physiological conditions, including depression, anxiety, dysfunctional and alexithymic psychological profile, pain, neuroticism and fibromyalgia [4,5]. Manzo et al. reported in 2019 a significant correlation between the fatigue severity and the presence of cognitive symptoms measured by objective cognitive tests, such as working and verbal memory dysfunction, attention disorders and alteration in executive functions [6]. 

However, psychological factors and profiles are not able to explain the markedly elevated prevalence of fatigue in patients with rheumatic diseases, implying the contribution of additional, still-unexplored molecular, genetic and metabolic factors to its development and regulation [5]. Therefore, fatigue is increasingly considered as a biological and brain phenomenon [7,8]. Autoantibodies are features of many rheumatic diseases, and several studies have explored a potential correlation between specific autoantibodies, fatigue and cognitive dysfunction. Most notably, a subset of pathogenic anti-double-stranded DNA (anti-dsDNA) antibodies cross-reacting with a single epitope present in GluN2 a/b subunits of the extracellular, ligand-binding domain of the N-methyl-D-aspartate receptor (NMDAR) have been identified as possible pathogenic factors closely associated with cognitive dysfunction, both in patients with SLE (systemic lupus erythematosus) und pSS (primary Sjögren’s syndrome) [9,10,11]. Although their prevalence is markedly elevated in SLE and pSS patients with neuropsychiatric manifestations, anti-NMDAR antibodies are not restricted in these populations. In particular, autoantibodies to NMDAR subunit NR1 or voltage-gated potassium channel complex have been associated with limbic encephalitis [12]. 

We recently identified a strong correlation of circulating antibodies to the NMDAR subunit NR2 with fatigue in SLE patients [11]. In the present study, we explored the presence of these anti-NMDAR antibodies in patients with other inflammatory and non-inflammatory rheumatic diseases suffering from fatigue. In addition, we investigated the association of these autoantibodies with circulating neurofilament level, as a marker of neuronal damage and blood–brain barrier (BBB) breach. 

## 2. Results

### 2.1. Demographic Characteristics of the Patients

Our cohort included 88 patients, 79 females and 9 males, with a mean age of 50.97 years and a mean of body-mass index (BMI) 26.29 kg/m^2^ and suffering from different grades of cognitive, motoric and total fatigue, according to the questionnaires of Penner et al. [13] (Table 1).

### 2.2. Clinical Characteristics of the Patients

Of the 88 patients included in this study, 23 patients were diagnosed with Sjögren’s syndrome, 32 patients with systemic lupus erythematosus and 14 patients with other autoimmune rheumatic diseases (limited and diffuse systemic sclerosis, polymyositis, rheumatoid arthritis, IgG 4 associated disease), as well as 7 patients with non-autoimmune inflammatory rheumatic disease (psoriatic arthritis, spondyloarthritis) and 12 patients with non-inflammatory rheumatic disease (osteoarthritis and fibromyalgia) (Table 2).

**Figure 1:**
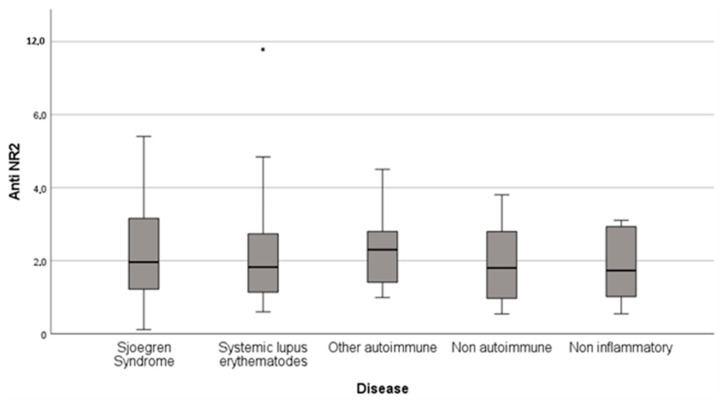
Distribution of anti-NR2 antibody titer in the different groups of fatigued patients with rheumatic diseases.

Elevated anti-NR2 antibodies (titer > 2 ng/mL) were presented in 48.8% (*n* = 43) of total fatigued rheumatic patients, in 47.8% (*n* = 11) of SS patients, 46.9% (*n* = 15) of SLE patients, 57.1% (*n* = 8) of patients with other autoimmune rheumatic disease, 42.9% (*n* = 3) of the patients with non-autoimmune inflammatory disease and 50% (*n* = 6) of the patients with non-inflammatory rheumatic disease (Table 2, Figure 1). 

Among all rheumatic patients (*n* = 88), 72.7% (*n* = 64) of the patients were graded as severely fatigued, 13.6% (*n* = 12) as moderately fatigued, 11.4% (*n* = 10) as mildly fatigued and 0.01% (*n* = 2) did not have fatigue based on the total FSMC score as determined by Penner et al., 2009.

The results for the different fatigue sub-categories were similar: 55.7% (*n* = 49) of the rheumatic patients suffered from severe cognitive fatigue, 79.5% (*n* = 70) severe motor, 22.7% (*n* = 20) moderate cognitive, 11.4% (*n* = 10) moderate motor, 12.5% (*n* = 11) mild cognitive, 4.5% (*n* = 4) mild motor, 9.1% (*n* = 8) of the patients did not suffer from cognitive fatigue and 4.5% (*n* = 4) did not suffer from motor fatigue.

### 2.3. Patients with Positive anti-NR2 Antibody Titer

Furthermore, we analyzed the distribution of the fatigue grade for particular fatigue categories (cognitive, motor and total fatigue) in the different groups of rheumatic patients with positive anti-NR2 titers. All patients (*n* = 43) with an anti-NR2 titer >2 ng/mL were included in further analysis. Anti-NR2 positive patients from all disease groups predominantly suffered from severe fatigue level regardless of the fatigue sub-categories.

**Figure 2:**
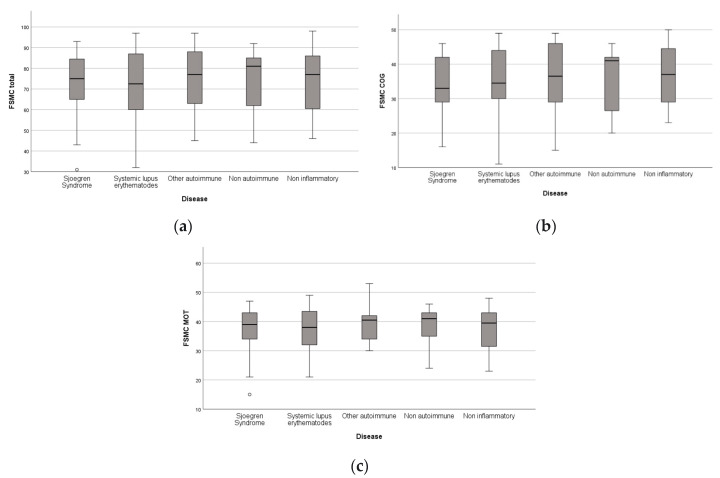
(**a**–**c**) Severity of total fatigue in patients with positive anti-NR2 titer according to the FMSC score.

When considering only the anti-NR2-positive patients, 81.8% (*n* = 9) of those with SS, 66.7% (*n* = 10) of those with SLE, 87.5% (*n* = 7) of those with other autoimmune rheumatic diseases, 66.7% (*n* = 2) of those with non-autoimmune inflammatory diseases and 83.3% (*n* = 5) of those with non-inflammatory rheumatic diseases suffered from severe total fatigue (Figure 2a). In terms of cognitive fatigue in the anti-NR2-positive patients, 36.4% (*n* = 4) of those with SS, 46.7% (*n* = 7) of those with SLE, 87.5% (*n* = 7) of those with other autoimmune rheumatic diseases, 67.7% (*n* = 2) of those with non-autoimmune inflammatory rheumatic diseases and 83.3% (*n* = 5) of those with non-inflammatory rheumatic diseases suffered from severe levels of cognitive fatigue (Figure 2b). Focusing on motor fatigue, 81.8% (*n* = 9) of the anti-NR2-positive SS patients, 93.3% (*n* = 14) of the positive SLE patients, 100% (*n* = 8) of the positive patients with other autoimmune rheumatic diseases, 66.7% (*n* = 2) of the positive patients with non-autoimmune inflammatory rheumatic diseases and 83.3 (*n* = 5) of the positive patients with non-inflammatory rheumatic diseases suffered from severe levels of motor fatigue (Figure 2c). 

Patients with rheumatic diseases and positive anti-NR2 titers suffered predominantly from severe fatigue. In particular, 76.7% (*n* = 33) of the anti-NR2 positive patients (*n* = 43) had total FSMC scores valued as severe, 88.4% (*n* = 38) of these patients had motor FSMC scores valued as severe and 58.1% (*n* = 25) of these patients had cognitive FSMC scores valued as severe. 

### 2.4. Correlation between Antibody Titer and Fatigue Severity 

No significant correlation between anti-NR2 antibody titer and fatigue severity could be detected over all the disease groups, except for cognitive fatigue in the group of patients with other autoimmune diseases (*n* = 14, r = 0.594, *p* ≤ 0.05) (Table 3(a)). However, with regard to the FSMC motor score (0.461, *p* = 0.097) and FSMC total score (0.490, *p* = 0.075) in this group of patients, no correlation was found (Table 3(b,c)).

### 2.5. Correlation of the Circulating NfL Level, Anti-NR2 Antibody Titer and Fatigue Severity in Patients with Rheumatic Diseases

The correlation analysis of circulating NfL level (pg/mL) and anti-NR2 antibody titer in patients from different disease groups revealed no significant correlation between the two biomarkers in patients with fatigue (Table 4). 

**Figure 3:**
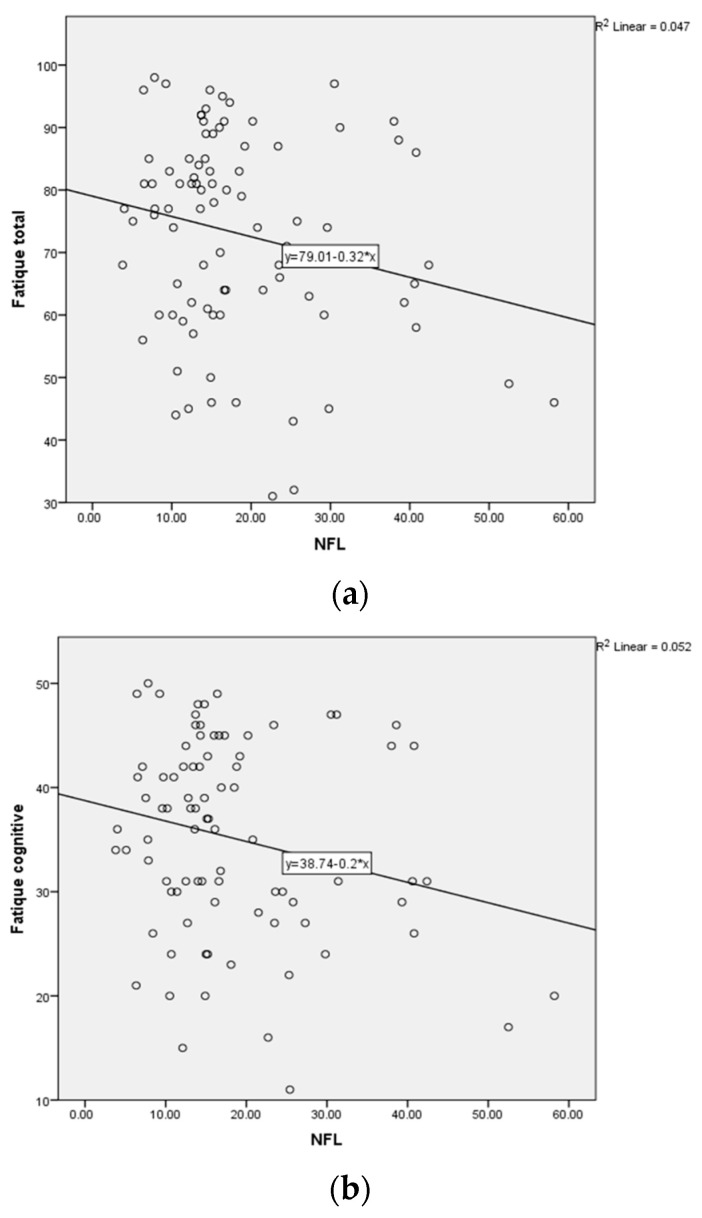

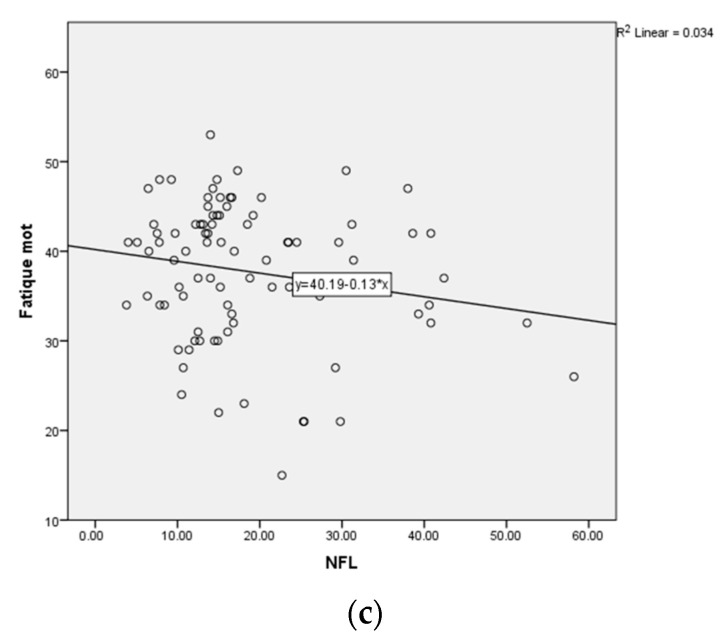
(**a**–**c**) Correlation by Spearman-Rho of circulating NfL level and fatigue severity in patients with positive anti-NR2 antibody titer (>2 ng/mL).

Furthermore, we correlated the circulating NfL level (pg/mL) with the fatigue severity, according to the FSMC scores for the different categories (cognitive, motor, total) in the patients with positive (>2 ng/mL) *n* = 43 and negative (<2 ng/mL) *n* = 45 anti-NR2 antibody titers. There was no significant correlation by Spearman-Rho for patients with positive anti-NR2 titer (>2 ng/mL) (*n* = 43) as well as for patients with negative anti-NR2 titer (<2 ng/mL) (*n* = 45) (Table 5, Table 6 and Table 7, Figure 3) concerning the FSMC cognitive, motor and total score.

There was no significant correlation by Spearman-Rho between the adjusted NfL level (Z-score NfL) in accordance with age and BMI of a representative collective regarding the FSMC cognitive score, the FSMC motor score and the FSMC total score (Table 8). 

Moreover, we correlated the circulating NfL level (pg/mL) with the fatigue severity, according to the FSMC scores in the different groups of patients. In accordance with the previous results, no correlations were detected.

### 2.6. Distribution of the Anti-NR2 Antibody Titer in Patients with Rheumatic Diseases

In addition, we analyzed the distribution of the anti-NR2 antibody titer in the population of patients with rheumatic diseases. 

**Figure 4:**
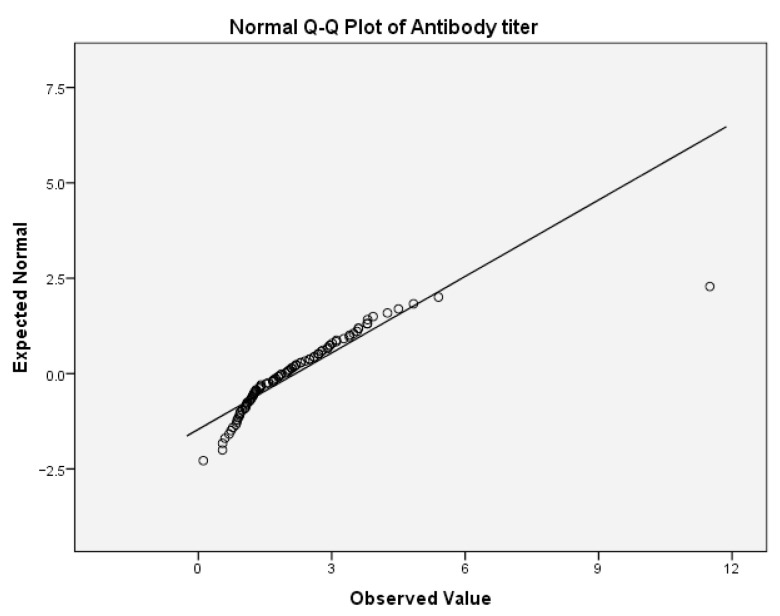
Normal Q-Q plot of anti-NR2 titer.

**Figure 5:**
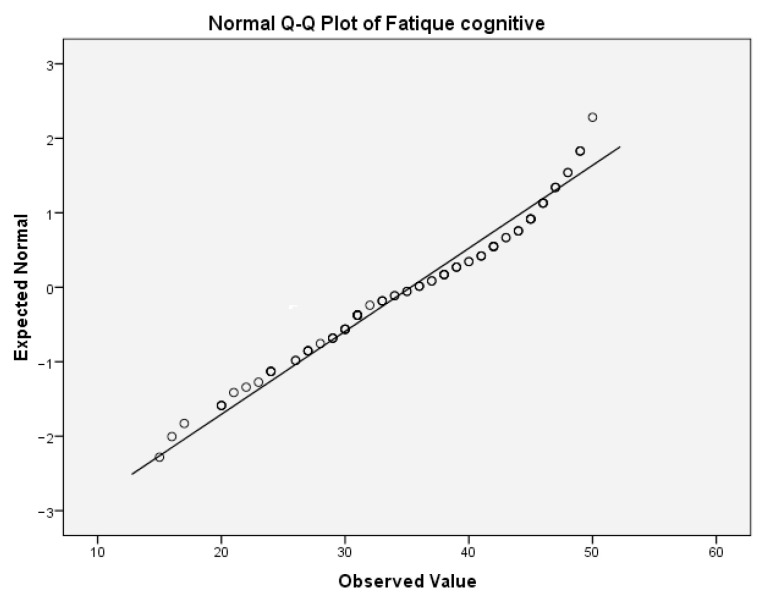
Normal Q-Q plot of cognitive fatigue.

First, we tested the variables antibody titer and cognitive fatigue for the normality of the distribution. For the antibody titer, the values for Skewness z-value and Kurtosis z-value were 11.63 and 31.93. Those values were outside the interval −1.96, 1.96 which indicates excessive deviation from the normal distribution. For the cognitive fatigue, the corresponding values for Skewness z-value and Kurtosis z-value were 1.53 and 1.21. Those values were in the corresponding interval of acceptable values −1.96, 1.96. Table 9(a) presents the test of normality for cognitive fatigue. In this table, the values of the Shapiro–Wilk test indicate that the significance for rejecting the null hypothesis is below the critical value of 0.05. Thus, both variables are not normally distributed. This can be also concluded from the following Q-Q boxplots (Figure 4 and Figure 5).

The same analyses were performed for the variable motor fatigue. For the motor fatigue, the values for Skewness z-value and Kurtosis z-value were 2.623 and 0.104, respectively. The Skewness z-value was outside the interval −1.96, 1.96, indicating that the values were not normally distributed. Table 9(b) provides the test of normality of this variable. 

**Figure 6:**
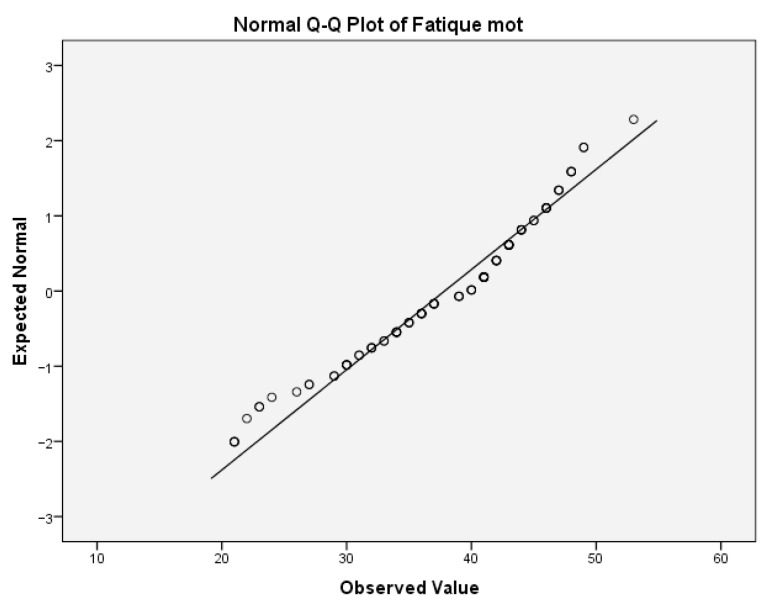
Normal Q-Q plot of motor fatigue.

The values of the Shapiro–Wilk test indicate that the significance for rejecting the null hypothesis was below the critical value of 0.05. Thus, the variable motor fatigue was not normally distributed. This can also be concluded from the boxplot diagram, which is given below (Figure 6).

For the variable total fatigue, there was no need to perform an analysis of normality of distribution. If the two variables, cognitive and motor fatigue, that are part of this variable do not have a normal distribution, then it can be safely said that this variable also does not have a normal distribution.

## 3. Discussion

NMDAR signaling is responsible for the majority of excitatory synaptic transmission in the CNS, as an elementary mechanism of synaptic plasticity, fundamental for learning and memory [10]. It affects almost all forms of brain activity, including those important for higher brain functions. It has been well established that anti-NR2 antibodies in the brain are related to the neurocognitive impairment in patients with neuropsychiatric SLE (De Giorgio et al.) [14]. Moreover, it has been shown that anti-NR2 antibodies are associated with depressive mood (Lapteva et al.) [15], as well as decreased short memory and learning abilities (Omdal et al.) [16]. In addition, Lauvsnes et al. found a correlation of anti-NR2 antibodies with hippocampal atrophy and cognitive impairment, not only in patients with SLE, but also in patients with pSS [17]. 

In our previous study we revealed an association of the circulating anti-NR2 antibodies and fatigue in patients with SLE. In the present study we report that circulating anti-NR2 antibodies are not exclusively present in fatigued patients with SLE, but can also be detected in fatigued patients with SS and other autoimmune rheumatic diseases (limited and diffuse systemic sclerosis, polymyositis, rheumatoid arthritis, IgG 4 associated disease). 

This distribution is in accordance with the pathogenetic and phenotypic similarities, as well as the frequent overlap of diseases in the group of autoimmune diseases. Thus, we can confirm the results of a recent meta-analysis (17 studies), describing increased anti-NMDAR antibody prevalence in SLE (24.6%) and Sjögren’s syndrome (19.7%) compared to healthy people (7.6%) [18]. However, a recent study in over 7000 subjects reported that seroprevalence, immunoglobulin class, or titers of serum antibodies against brain-antigens (including anti-NMDAR) did not predict disease [19]. 

Additionally, we found positive anti-NR2 antibody titers in patients with autoinflammatory rheumatic diseases (PsA, SpA); however, the prevalence of positive titers in this patient group seems to be lower. 

These findings suggest an individual role of anti-NR2 antibodies in the pathophysiology of fatigue, regardless of the main disease. Therefore, it could be postulated that the detection of anti-NR2 antibodies may represent a potential biomarker of fatigue, which is disease independent.

Although we could not find a significant correlation between the fatigue severity and the titer of anti-NR2 antibodies, the results of this study demonstrate that rheumatic patients with positive anti-NR2 titers suffer predominantly from severe fatigue in all fatigue categories.

Numerous factors can influence the plasma anti-NR2 antibody concentration and thereby obscure the understanding of how the circulating levels reflect the pathophysiology and clinical and laboratory findings [20]. It is still a matter of debate whether elevation of anti-NR2 antibodies in the cerebrospinal fluid (CSF) of patients with autoimmune diseases is due to increased intrathecal synthesis or to a transport from the peripheral blood circulation to the CSF through a damaged BBB [11]. Kowal et al. found in 2016 that mice with high levels of anti-NR2 antibodies have no neuronal damage until BBB breakdown takes place [21]. Apparently, an intact BBB prevents the transport of anti-NR2 from the systemic circulation into the brain [20]. Thus, the levels of anti-NR2 antibodies in CSF are more likely to distinguish between patients with or without neuro-cognitive manifestations, including fatigue, than the circulating anti-NR2 levels. However, repeated collections of CSF to monitor fatigue are invasive and cannot be performed on regular basis, and thus we considered this infeasible in our patient population. Only blood-based measures of anti-NR2 antibodies would be convenient for the population of fatigued patients und could offer a broad application in clinical routine. 

For the purpose of providing information equivalent to CSF testing, via blood-based measures, we used a combination of a circulating anti-NR2 antibody titer and circulating NfL level as a composite diagnostic. Secondly, we explored the correlation between anti-NR2 antibody titers, as brain-reactive autoantibodies and circulating NfL levels, as a surrogate biomarker in rheumatic patients with fatigue. NfL is a structural protein of the neuronal cytoskeleton, exclusively expressed in central and peripheral neurons [22]. Following neuronal damage due to neurodegenerative, inflammatory, vascular or traumatic processes, these proteins are released into CSF and consecutively into the blood to a lesser extent [23]. Accordingly, NfL is a promising biomarker for neuronal damage in neurodegenerative conditions, multiple sclerosis, cardiovascular diseases, and traumatic brain injury and can predict future rates of cognitive decline [24]. In addition, NfL levels in the CSF (CSF NfL) correlate with central nervous system (CNS) involvement in autoimmune inflammatory diseases, such as multiple sclerosis, SLE and primary Sjögren’s syndrome [25,26]. In general, NfL level increases are unspecific, as they may arise from any process resulting in neural damage, which is often viewed as an obstacle for the implementation of sNfL level assessment into clinical practice. However, fatigue is not restricted to specific pathophysiological processes, and this lack of specificity might actually be beneficial because it enables the detection of a wide range of potential neuronal damages with diffuse or polytope localization in the brain [23]. Tjensvoll et al. reported in 2021 that increasing concentrations of NfL in CSF were associated with increasing levels of anti-NR2 antibodies in CSF and reflected cognitive dysfunction in patients with SLE and pSS [24]. Moreover, they found no differences in NfL concentrations between patients with SLE und pSS, indicating that the neuronal pathogenetic impact of anti-NR2 antibodies is more or less similar in the two diseases [24]. Nevertheless, several studies suggest that plasma NfL could provide adequate clinical information, equivalent to CSF measures [23,25]. Plasma NfL can predict cognitive decline and changes in hippocampal volumes and fractional anisotropy in the corpus callosum [25]. Lauvsnes et al. found an association between plasma NfL concentrations in patients with SLE and some abnormal neurological, cognitive and neuroimaging findings [25]. Furthermore, they observed moderate correlation between CSF and plasma NfL concentrations [25]. Engel et at. reported increased sNfL levels in SLE patients with focal CNS involvement, whereas sNfL levels of SLE patients with diffuse CNS and peripheral nervous system involvement did not differ from those of SLE patients without neuropsychiatric manifestations [23]. 

However, in the present study, we did not find a significant correlation between anti-NR2 antibody titers as brain-reactive autoantibodies and circulating NfL levels in rheumatic patients with fatigue. These results are in accordance with the findings of Lauvsnes et al., who reported the lack of association between anti-NR2 antibodies and plasma NfL [25]. Moreover, there was no correlation between circulating NfL level and clinical fatigue level in rheumatic patients with positive anti-NR2 titers, suggesting that, not only neuronal damage and BBB integrity, but also other factors may be involved in the pathophysiology of anti-NR2 antibodies. 

The present study has some limitations. First, the anti-NR2-antibody titers were categorically defined as positive and negative using the cut-off for the ELISA assay. The data of normal healthy individuals who tested negative for anti-NMDA receptor antibodies by ELISA are needed to assess their cut-off values precisely and interpretate the clinical results more accurately. A second limitation was the small cohort of patients with rheumatic diseases and the different number of participants in each subgroup. Lastly, the investigations of anti-NMDA receptor antibodies and NfL were only performed on blood samples and did not include CSF samples or results of imaging techniques such as cMRT.

Experimental studies are not concordant to a single theory with regard to the pathophysiological mechanism of anti-NR2 antibodies. Besides the theory of neuronal apoptosis via excitotoxicity, there are suggestions of alternative hypotheses, including reduced energy metabolism and immune-metabolic disturbances [11].

The mechanisms of pathogenicity could be decisive for the degree of recovery of brain function. Whereas the excitotoxic cell lysis results in irreversible tissue destruction, a pathogenic effect caused by cell signaling alterations and internalization of membrane receptors can be reversed upon removal of antibodies [25,27,28]. 

Overall, further studies are required to investigate the anti-NR2 pathophysiology and reveal if the cerebral manifestations related to the anti-NR2 antibodies are potentially reversible or static due to a neuronal death. This would offer new approaches in the therapy of the clinical conditions mediated by these antibodies. 

## 4. Materials and Methods

### 4.1. Study Design and Patient Cohort 

Between 2020 and 2022, patients treated at the Center for Rheumatic Diseases Rhineland-Palatinate GmbH, Bad Kreuznach (Germany) were screened for eligibility for inclusion in this prospective study based on available medical records. Inclusion criteria were a confirmed rheumatologic diagnosis from a rheumatologist, as well as presence of fatigue according to the FSMC (fatigue scale for motor and cognitive functions) questionnaire.

Clinical, histological, laboratory, psychometric and personality data were also collected.

Written informed consent was obtained from all patients.

### 4.2. Analysis of Anti-NR2 Antibody and sNfL Levels

Freshly drawn blood samples were collected, centrifuged and stored at −80 °C. For the detection of circulating anti-NR2 antibodies, human serum was analyzed using an enzyme-linked immunosorbent assay (ELISA)(NR2AT-IFA, Cat. No. ENA100, DRD Biotech) according to the manufacturer’s instructions. All samples were analyzed retrospectively.

Anti-NR2 titers >2 ng/mL were considered as elevated or positive, according to the ELISA Kit manufacturer’s instructions.

Analysis of serum neurofilament light chain (sNfL) levels were determined using highly sensitive single molecule array (SiMoA, Billerica, MA, USA) technology. Samples were measured in duplicate in several rounds by SiMoA HD-1 (Quanterix, Billerica, MA, USA) using NF-Light Advantage Kits (Quanterix) [19] according to the manufacturer’s instructions. Resorufin-β-D-galactopyranoside (RGP) was incubated at 33 °C for 60 min prior to running the assay. sNfL measurements were performed in a blinded fashion without information about clinical data. sNfL Z scores were derived from a recently published database comprising 4532 control persons with no evidence of CNS disease taking part in four cohort studies in Europe and North America (Benkert et al., Lancet Neurol 2022) [29]. The authors of this study modelled the distribution of sNfL concentrations in the function of physiological age-related increase and body-mass index (BMI)-dependent modulation, to derive Z score values from this reference database, via a generalized additive model for location, scale and shape. The application we used to calculate age- and BMI-adjusted sNfL Z scores based on this reference database can be accessed at the following link: http://shiny.dkfbasel.ch/baselnflreference (accessed on 27 July 2022).

### 4.3. Fatigue Status

In parallel, the degree of fatigue for motor and cognitive functions was recorded using the FSMC (fatigue scale for motor and cognitive functions) questionnaire [13]. The FSMC contains 20 items. Ten items measure symptoms of motor and cognitive fatigue. Responses are scored on a five-point scale. Item scores can be summed to a global severity score for FSMC-total or for the two sub-scales FSMC-cognitive and FSMC-motor. 

This questionnaire enables us to differentiate cognitive from motor fatigue and to stratify the degree of severity into 4 groups. While the FSMC questionnaire was originally developed for patients with Multiple Sclerosis (MS), we have adapted it for patients with rheumatic diseases. 

### 4.4. Statistical Analyses

All data were assessed for normal or non-normal distribution. Differences in disease scores were determined using the Mann–Whitney U and H-Test. Correlations were determined by Spearman’s Rho correlation analysis. The level of significance was set at α = 0.05. The resulting *p*-values were considered nominally significant at *p* < 0.05. Statistical analyses were calculated with SPSS PASW27 Statistics (IBM Corp., Somers, NY). Figures were created using GraphPad Prism for Windows (Microsoft, Redmond, WA, USA).

## 5. Conclusions

Fatigue is a widespread and complex symptom that is diagnosed by questionnaire. Here, we report that the presence of circulating anti-NR2 antibodies is associated with severe fatigue in patients with both autoimmune and non-autoimmune rheumatic diseases. Thus, the detection of these autoantibodies might be a helpful diagnostic tool in rheumatic patients with fatigue. 

Furthermore, the presence of anti-NR2 antibodies in different subsets of rheumatic patients, independently from the main disease, suggests an individual role of these autoantibodies in fatigue pathophysiology.

The lack of correlation between the circulating NfL levels, anti-NR2 antibody titer and clinical fatigue manifestations indicates alternative factors and theories in fatigue pathophysiology, such as chronification and microglial activation.

Further studies on a larger cohort are needed to confirm the validity of anti-NR2 antibodies as a biomarker of fatigue in patients with rheumatic autoimmune diseases. 

## Figures and Tables

**Table 1:** Demographic characteristics of the patients.

	*n*	Mean Value	Std. Deviation	Minimum	Maximum
Gender					
Female (%)	79 (89.77)				
Male (%)	9 (10.23)				
Age	88	50.97	13.26	21	81
BMI	88	26.29	6.39	15.89	53.22
FSMC total	88	73.02	16.25	31	98
FSMC cognitive	88	35.12	9.30	11	50
FSMC motoric	88	37.76	7.73	15	53
Anti NR2	88	2.19	1.50	0.11	11.50

**Table 2:** Clinical characteristics of the patients and distribution of positive anti-NR2 antibody titer in the different groups of fatigued patients with rheumatic diseases.

Disease Group	Diagnosis	N	Positive Anti-NR2 Antibody Titer (>2 ng/mL)
**Sjögren Syndrome**	Sjögren Syndrome	23	(*n* = 11) 47.8%
**Systemic lupus erythematosus**	Systemic lupus erythematosus	32	(*n* = 15) 46.9%
**Other autoimmune rheumatic disease**	Systemic sclerosis, Mixed connective tissue disease, Polymyositis, Rheumatoid arthritis, IgG 4 associated disease	14	(*n* = 8) 57.1%
**Non-autoimmune inflammatory rheumatic disease**	Psoriatic arthritis, Ankylosing Spondylitis, Peripheral Spondyloarthritis	7	(*n* = 3) 42.9%
**Non-inflammatory rheumatic disease**	Fibromyalgia, Osteoarthritis	12	(*n* = 6) 50.0%
**Total**		**88**	**43 (48.8%)**

**Table 3:** (**a**) Correlation by Spearman-Rho of circulating anti-NR2 antibody titer and cognitive fatigue severity in the different groups of patients with rheumatic diseases. (**b**) Correlation by Spearman-Rho of circulating anti-NR2 antibody titer and motor fatigue severity in the different groups of patients with rheumatic diseases. (**c**) Correlation by Spearman-Rho of circulating anti-NR2 antibody titer and total fatigue severity in the different groups of patients with rheumatic diseases.

	*n*	r, Correlation Coefficient	*p*, Probability Value
(**a**)			
SS	23	0.255	0.240
SLE	32	0.229	0.208
Other autoimmune	14	0.549	<0.05
Non-autoimmune inflammatory	7	0.255	0.582
Non-inflammatory	12	0.364	0.24
(**b**)			
SS	23	0.215	0.325
SLE	32	0.085	0.643
Other autoimmune	14	0.461	0.097
Non-autoimmune inflammatory	7	0.126	0.788
Non-inflammatory	12	0.364	0.244
(**c**)			
SS	23	0.232	0.287
SLE	32	0.066	0.721
Other autoimmune	14	0.490	0.075
Non-autoimmune inflammatory	7	0.162	0.728
Non-inflammatory	12	0.371	0.235

**Table 4:** Correlation by Spearman-Rho of circulating NfL level and anti-NR2 antibody titer in the different groups of patients with rheumatic diseases.

	*n*	r, Correlation Coefficient	*p*, Probability Value
SS	23	0.190	0.386
SLE	32	0.035	0.851
Other autoimmune	14	0.051	0.864
Non-autoimmune inflammatory	7	−0.464	0.294
Non-inflammatory	12	0.252	0.429
Total	88	0.067	0.534

**Table 5:** Correlation by Spearman-Rho of circulating NfL level and fatigue severity in patients with positive anti-NR2 antibody titer (>2 ng/mL) *n* = 43.

*n* = 43	r, Correlation Coefficient	*p*, Probability Value
FSMC cognitive	−0.160	0.305
FSMC motor	−0.158	0.310
FSMC total	−0.152	0.329

**Table 6:** Correlation by Spearman-Rho of circulating NfL level and fatigue severity in patients with negative anti-NR2 antibody titer (<2 ng/mL) *n* = 45.

*n* = 45	r, Correlation Coefficient	*p*, Probability Value
FSMC cognitive	−0.133	0.385
FSMC motor	−0.050	0.745
FSMC total	−0.107	0.485

**Table 7:** Correlation by Spearman-Rho of circulating NfL level and fatigue severity in all patients with rheumatic diseases *n* = 88.

*n* = 88	r, Correlation Coefficient	*p*, Probability Value
FSMC cognitive	−0.150	0.163
FSMC motor	−0.093	0.389
FSMC total	−0.118	0.272

**Table 8:** Correlation by Spearman-Rho of adjusted NfL level (Z-score) and fatigue severity in all patients with rheumatic diseases *n* = 88.

*n* = 88	r, Correlation Coefficient	*p*, Probability Value
FSMC cognitive	−0.127	0.239
FSMC motor	−0.097	0.370
FSMC total	−0.127	0.239

**Table 9:** (**a**) Tests of normality for cognitive fatigue. (**b**). Tests of normality for motor fatigue.

	Kolmogorov–Smirnov ^a^			Shapiro–Wilk		
	Statistic	Df	Sig.	Statistic	Df	Sig.
(**a**)						
Antibody titer	0.124	88	0.002	0.777	88	0.000
Fatigue cognitive	0.088	88	0.086	0.965	88	0.018
(**b**)						
Fatigue motor	0.140	88	0.000	0.958	88	0.007

^a^ Lilliefors Significance Correction.

## Data Availability

The data sets used and/or analyzed during the current study are available from the corresponding author upon reasonable request.
